# Beyond TCR Signaling: Emerging Functions of Lck in Cancer and Immunotherapy

**DOI:** 10.3390/ijms20143500

**Published:** 2019-07-16

**Authors:** Ursula Bommhardt, Burkhart Schraven, Luca Simeoni

**Affiliations:** Institute of Molecular and Clinical Immunology, Otto-von-Guericke University, Leipziger Strasse 44, 39120 Magdeburg, Germany

**Keywords:** Lck, T cell, CAR, cancer, leukemia, brain

## Abstract

In recent years, the lymphocyte-specific protein tyrosine kinase (Lck) has emerged as one of the key molecules regulating T-cell functions. Studies using Lck knock-out mice or Lck-deficient T-cell lines have shown that Lck regulates the initiation of TCR signaling, T-cell development, and T-cell homeostasis. Because of the crucial role of Lck in T-cell responses, strategies have been employed to redirect Lck activity to improve the efficacy of chimeric antigen receptors (CARs) and to potentiate T-cell responses in cancer immunotherapy. In addition to the well-studied role of Lck in T cells, evidence has been accumulated suggesting that Lck is also expressed in the brain and in tumor cells, where it actively takes part in signaling processes regulating cellular functions like proliferation, survival and memory. Therefore, Lck has emerged as a novel druggable target molecule for the treatment of cancer and neuronal diseases. In this review, we will focus on these new functions of Lck.

## 1. Introduction

The lymphocyte-specific protein tyrosine kinase (Lck) is a member of the Src family of protein tyrosine kinases firstly identified in the 1980s [1,2]. Since then, the function of Lck has been extensively investigated and many mechanistic insights into the regulation of its activity have been revealed. Nevertheless, Lck remains in many ways an enigmatic protein and recent research has provided new insights into the function of Lck in T-cell activation mediated by chimeric antigen receptors (CARs), in signal transmission in cancer cells, and also in the brain. In this article, we summarize recent advances on Lck biology in TCR signaling and on its role in the new emerging research fields.

## 2. Regulation of Lck Activation

**Figure 1:**
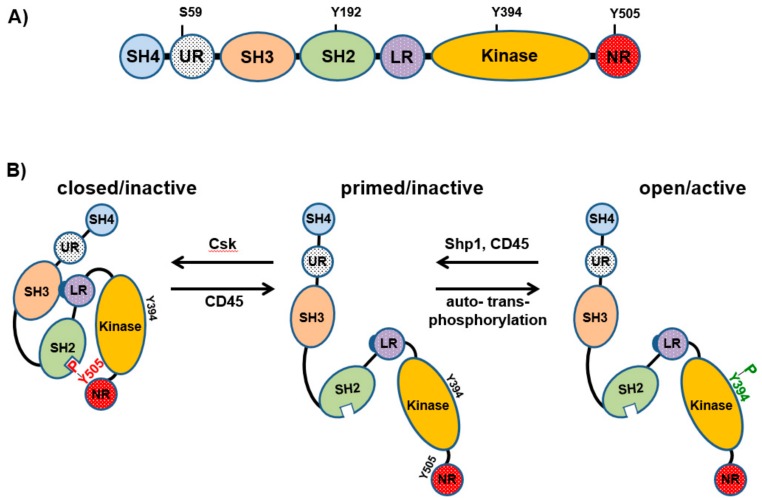
Representation of Lck structure and regulation of its kinase activity. (**A**) Schematic structure of Lck. SH4, unique region (UR), SH3, SH2, SH2-kinase domain linker region (LR), kinase domain, and the C-terminal negative regulatory tail (NR) are shown. Potential regulatory sites are indicated. (**B**) Lck conformations and regulation of Lck activation. Phosphorylation of the inhibitory tyrosine (Y505) by Csk results in a close/inactive conformation. Dephosphorylation of Y505 by CD45 primes the enzyme. Auto- trans-phosphorylation of Y394 results in an open/active conformation. Dephosphorylation of Y394 by phosphatases reverts active Lck back to the primed conformation.

From the structural point of view, Lck has the typical organization found in all members of the Src kinase family comprising an N-terminal site (Src-homology 4, SH4 domain), a unique region, a SH3 and SH2 domain, a catalytic domain, and a short C-terminal tail (Figure 1A) [3]. The SH4 domain contains a glycine and two cysteine residues, which are myristoylated and palmitoylated, respectively. These lipid modifications are required to target Lck to the plasma membrane. On the other hand, the SH3 and SH2 domains mediate inter- and intramolecular interactions, which are important for the regulation of Lck activity and signal transmission.

The activity of Lck is regulated via phosphorylation/dephosphorylation of crucial tyrosine residues, as well as by conformational changes (Figure 1B). Phosphorylation of Y505 by the C-terminal Src kinase (Csk) results in an intramolecular interaction with the SH2 domain, which in turn closes Lck. The closed conformation of Lck is further stabilized by the interaction between the SH3 domain and a proline-reach region located in the SH2-kinase domain linker. Dephosphorylation of Y505, which is primarily mediated by the protein tyrosine phosphatase CD45, opens Lck. Subsequently, open Lck auto- and transphosphorylates Y394, which is located in the activation loop within the catalytic domain, thus resulting in full activation of Lck.

In addition to Y394 and Y505, the activity of Lck is regulated by phosphorylation of other amino acid residues (Figure 1A). Recently, by using a phosphomimetic Lck mutant, a study has shown that phosphorylation of Y192 within the SH2 domain may prevent the interaction between Lck and CD45, thus resulting in hyperphosphorylation of Y505 and consequently in the inactivation of Lck [4]. It has been further proposed that the phosphorylation of this site is Zap-70-dependent and that Y192 is part of an inhibitory feedback loop, which may be important for the regulation of the amount of active Lck and the strength/duration of TCR signaling [5]. In addition to Y192, S59, located in the unique domain of Lck (Figure 1A), is part of another feedback circuit required for the regulation of TCR signaling. The data suggest that S59 is phosphorylated by the extracellular signal-regulated kinases (Erk1/2) [6], whereas the phosphatase Calcineurin dephosphorylates S59 [7]. However, whether phosphorylation of S59 positively or negatively regulates Lck activity is still controversial. Initial observations indicated that phosphorylation of S59 reduces Lck activity [8]. Recent data have corroborated this hypothesis showing that TCR signaling is inhibited in Jurkat T cells expressing a phosphomimetic S59E Lck mutant [7]. Conversely, another study has shown that phosphorylation of S59 impairs the interaction between Lck and the phosphatase SHP-1, thus preventing dephosphorylation of Y394 and Lck inactivation in thymocytes [9]. In agreement with these findings, we have found that blockade of the Erk1/2-Lck feedback loop and hence S59 phosphorylation rapidly terminates proximal TCR signaling [10].

Collectively, the data discussed above indicate that the activity of Lck is tightly regulated by a number of biochemical modifications, conformational dynamics, and signaling circuits. The reason for this complex regulation may be explained by the fact that Lck is crucial for the initiation of proximal signaling events downstream of the TCR and consequently for T-cell activation and all T cell responses (see chapter 3). In addition, deregulation of Lck activity may also play an important role in cellular transformation (see chapters 5 and 6) [11,12].

## 3. The Role of Lck in T-Cell Activation

According to a well-accepted view, engagement of the TCR by agonist ligands results in the activation of Lck. Nevertheless, a clear demonstration that Lck is indeed auto- and trans-phosphorylated upon TCR triggering was missing until recently. Using an Lck biosensor and FRET/FLIM measurements together with biochemical analyses, we have shown that a fraction of Lck (about 20%) undergoes opening and de novo phosphorylation upon TCR stimulation [13]. Additional studies from our group have further demonstrated that Lck phosphorylation on the activatory Y394 is mandatory for the initiation of TCR signaling [14]. Our findings were most recently corroborated by an independent study using an elegant optogenetic system, which demonstrated that auto-phosphorylation of Y394 is indispensable for Lck catalytic activity and that Lck can stimulate its own activation by adopting a more open conformation [15]. Nevertheless, how Lck initiates TCR signaling is still a matter of debate [16,17]. In fact, an alternative model postulates that a pool of activated Lck, which is present in resting T cells and constitutes about 40% of total Lck, is sufficient for T-cell activation and that the levels of Lck phosphorylated on Y394 do not change upon TCR stimulation [18].

The activity of Lck is required for the phosphorylation of tyrosine residues within immunoreceptor tyrosine-based activation motifs (ITAMs) of the ζ-chains of the TCR/CD3 complex, which become available following a change in the conformation of the TCR upon engagement with its ligand [19]. How Lck is recruited to the activated TCR to phosphorylate the ITAMs has been extensively investigated during the past decades. Since Lck is known to be associated to the CD4/CD8 coreceptors [20], it has been postulated that the coreceptors are responsible for the targeted delivery of its associated Lck to the engaged TCR upon coreceptor binding to the MHC [21]. However, Lck exists either associated with the CD4/CD8 coreceptors or free [22,23,24]. While all Lck molecules are associated with the CD4/CD8 coreceptors in thymocytes [23], it has been shown that the coreceptors are not essential for the activation of mature T cells and that free Lck molecules initiate phosphorylation of the TCR/CD3 complex [25,26]. Therefore, in order to initiate signaling, free Lck should be recruited to the activated TCR. A study has recently assessed this question. The results of this work suggest that a basic residue-rich sequence in CD3ε ionically interacts with an amino acid stretch enriched of acidic residues in the unique domain of Lck and that this interaction is required to recruit the kinase to the TCR/CD3 complex and hence to initiate phosphorylation of the ITAMs [27].

ITAM phosphorylation is followed by the recruitment of the Syk family kinase Zap-70 to the engaged TCR via its tandem SH2 domain. Zap-70 in turn is fully activated upon Lck-mediated phosphorylation. The signal is further propagated by Zap-70, which phosphorylates the transmembrane adaptor protein linker for activation of T cells (LAT). LAT couples the TCR to the activation of intracellular signaling pathways, which ultimately culminates in transcriptional activation and T-cell proliferation (for reviews on T-cell activation see references [28,29,30]).

A recent study has shown that Lck plays an important role in the phosphorylation of LAT by acting as a molecular bridge [31]. According to this scenario, Lck facilitates the phosphorylation of LAT by Zap-70 by concomitantly binding ZAP-70 via its SH2 domain and LAT upon the interaction between the SH3 domain of Lck and a newly discovered conserved proline-rich motif in LAT. In addition to the proline-rich motif, LAT possesses another binding motif for Lck, which is enriched in negatively charged amino acids [32]. It has been proposed that binding of Lck to LAT via this motif negatively affects Lck kinase activity. In fact, expression of a LAT mutant in which the negatively charged amino acid was substituted enhanced proximal TCR signaling in J.CaM2 cells [33]. Thus, LAT also appears to play a role in the regulation of proximal TCR signaling.

In addition to its crucial function downstream of the TCR, Lck is also important for signal transmission by the costimulatory molecule CD28 to drive full T-cell activation (for a review see [34]). Lck phosphorylates tyrosine residues located in two signaling motifs within the cytosolic domain of CD28, YMNM and PYAP. Phosphorylation of the YMNM motif allows recruitment of the adaptors Grb2 and GADS and the p85 subunit of phosphoinositide 3-kinase (PI3K) to CD28, thus in turn linking CD28 to the activation of downstream signaling pathways such as Ras, Rac, and PI3K-Akt. The function of the PYAP motif appears to be more complex and partially unclear. It is thought that Lck directly binds to PYAP in a phosphorylation-independent manner via its SH3 domain. CD28-bound Lck may in turn phosphorylate PDK1, which is required for the activation of PKCθ, a crucial signaling effector downstream of CD28 that contributes to the activation of the transcription factor NF-κB. However, a recent work has shown that Lck binds to the phosphorylated PYAP motif via its SH2 domain [35]. In this scenario, the SH3 domain of Lck would be free to interact with a proline-rich domain of PKCθ and hence Lck could play a role in the recruitment of PKCθ to CD28 in the proximity of PDK1 [36]. CD28-mediated signaling has recently been proposed to be inhibited via dephosphorylation of the YMNM motif by Shp1/Shp2, which are recruited to PD-1 upon its ligation [37]. Lck functions also downstream of other receptors including Fas [38], β1-integrins [39], and K_v_1.3 potassium channels, which are recruited into the immunological synapse upon TCR stimulation [40].

## 4. Lck Function in CAR T Cells

Because of its central role in TCR and CD28 signaling, the recruitment and activation of Lck in signaling modules of chimeric antigen receptor (CAR)-engineered T cells have become a major research focus (Figure 1). CARs represent one of the therapeutic strategies to reprogram T cells to fight against tumors. CARs are synthetic constructs that can bind to target cell surface antigens (usually expressed on tumors) in an MHC-independent manner through a single-chain variable fragment (scFv) recognition domain [41]. This extracellular part is followed by a transmembrane domain and a cytosolic region containing signaling endodomains of the CD3-ζ chain, CD28, CD137, and ICOS, which are required to provide the CAR with all the signaling modules needed to drive full T-cell activation [42].

CAR T-cell therapies generate high rates of response and remission in cancer therapy, including acute and chronic leukemia and refractory B cell lymphomas [43]. In contrast to hematopoietic cancers, treatment of solid tumors with CAR T cells has been so far less successful [44]. This is mainly due to the lack of common antigens expressed by solid tumors, the complex and inhibitory tumor environment, and the presence of immunosuppressive CD4^+^Foxp3^+^ regulatory T cells (Treg) and other cells, which hamper the potency and persistence of CAR T cells. One additional problem in cancer immunotherapy is that CAR T cells themselves can enhance Treg potency by providing IL-2 for Treg consumption [42]. Therefore, regimens to enhance the efficacy of CAR T cells in killing solid tumor cells include lymphodepletion (which, however, is non-specific, toxic and only leads to transient depletion of Treg cells) and modification of IL-2 production by CAR T cells. Regarding the latter point, deletion of the Lck binding motif within the CD28 endodomain of CARs has been shown to abrogate IL-2 induction upon CAR ligation and to improve anti-tumor activity of CAR T cells even in the presence of Treg cells [45,46]. Opposite effects were observed when the Lck-binding motif of CD28 was mutated in tumor models in which TGFβ, which accumulates in the tumor tissue, was the factor mediating T-cell suppression. In these models, resistance to TGFβ-mediated CAR T-cell suppression required an intact Lck-binding motif and IL-2 production [47].

Whereas the effector function of CAR T cells has been investigated in many tumor models, the signaling mechanisms provided by CARs are less well understood. Analysis of immunological synapse formation in dual-specific CAR T cells, where the same CTL was either triggered through the TCR or a CAR, revealed that the CAR-CTL synapse was less dependent on LFA1 for the formation of stable contacts with the target cell and that CAR-CTL receptors formed multifocal microclusters with Lck. CAR-CTLs also showed faster signaling, resulting in a quicker recruitment of lytic granules to the immune synapse and an accelerated detachment from the target cells [48]. Thus, CARs seem to form non-classical but potent immune synapses, apparently making CAR-driven T cells more potent serial killers of tumor cells than TCR-driven CTLs. Comparison of human CARs carrying different costimulatory sequences (CD3ζCD28 vs. CD3ζ-4-1BBζ), which are both effective against B cell malignancies but differ in effector function, toxicity and clinical efficacy, showed that stimulation of CD3ζCD28 CAR T cells leads to a faster and stronger protein phosphorylation and an effector T cell-like signaling signature [49]. This partly resulted from constitutive association of Lck with the CD28 endodomain (containing the PYAP motif) of the CAR, as signaling strength was reduced upon the mutation of this signaling domain. On the other hand, signaling induced by CD3ζ-4-1BBζ-CARs resulted in the induction of T cell memory type genes and a prolonged antitumor activity *in vivo*. These initial reports revealed that signaling domains involved in Lck binding are important factors in the regulation of CAR T-cell function. Further understanding of the signaling events induced by various CARs will be helpful in the development of more effective CAR modules.

## 5. Expression and Function of Lck in Leukemic Cells

In addition to T cells, Lck is expressed in NK T cells [50], NK cells [51,52], CD5^+^ B-1 B cells [53], germinal center and to a lesser extent in mantle zone B cells [54], and in aryl hydrocarbon receptor-activated primary human B cells [55]. The function of Lck in B-cell subsets remains unclear, as it has been proposed that Lck can both potentiate and suppress BCR signaling [56,57] and also inhibit IgM production [55]. Many years ago, Lck expression was also reported in lymphocytic leukemia of the B-cell lineage such as chronic lymphocytic leukemia (CLL) [58,59] and acute myeloid leukemia (AML) [60]. In the next section, we will summarize the current knowledge on the role of Lck in signaling processes in leukemic cells.

### 5.1. Chronic Lymphocytic Leukemia (CLL)

CLL, a heterogeneous hematopoietic malignancy of mature B cells, represents the most common form of leukemia among elderly adults in western countries, which, in its progressive form, is associated with serious morbidity and mortality [61]. One of the pathological features of CLL cells, which appears to be associated with the pathogenesis of the disease, is the continuous BCR signaling which regulates the survival and expansion of CLL cells [62]. Interestingly, CLL cells from different patients express a highly similar or ‘stereotypic’ BCR [63], which has been proposed to drive B-cell expansion upon recognition of an auto-antigen or antigen [62]. Consistent with the constitutive BCR signaling, CLL cells express higher levels of signaling proteins including Lyn, Syk, Btk, PLC-γ2, STAT3, and Erk1/2 [64,65,66,67]. As mentioned above, CLL cells express molecules which are normally involved in TCR signaling such as Lck and Zap-70 [68]. Because both Lck and Zap-70 are physiologically expressed in some B-cell subpopulations [53,54,69,70,71,72], it is still under debate whether Lck and Zap-70 are ectopically expressed in CLL or whether Lck- and Zap-70-positive CLL arise following transformation of B-cell subpopulations normally expressing these molecules. A study has shown that Lck expression in CLL cells is under the control of the transcription factor NFAT2 [73].

The pathogenic role of Zap-70 in CLL cell signaling has been extensively investigated because the expression of Zap-70 in CLL is strongly associated with an aggressive clinical course of the disease and poor prognosis [74]. It has been found that Zap-70 expression enhances BCR-mediated Erk1/2 and Akt activation, which in turn enhance proliferation and survival of CLL cells [75,76,77]. Moreover, Zap-70 upregulates CCR7 expression and increases CCR7- and CXCR4-mediated inside-out signaling to integrin activation, thus resulting in an enhanced response to chemokines and an increased migration of CLL cells [75,78]. However, it has also been proposed that the kinase activity of Zap-70 is not required for signal transmission in malignant B cells and that Zap-70 might act as an adaptor protein to facilitate BCR signaling in CLL cells [79,80].

**Figure 2:**
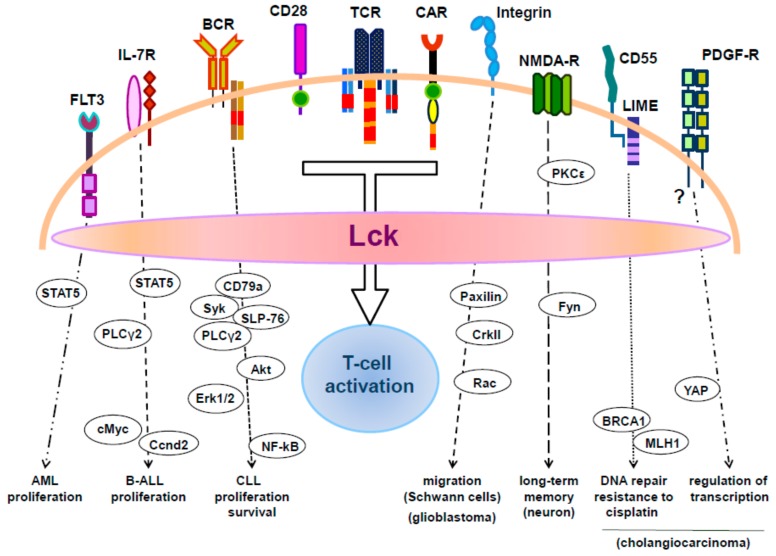
Schematic representation of Lck signaling and functions. Lck is expressed in different cell types and activates signaling pathways downstream of a variety of receptors regulating a multitude of cellular responses.

Conversely to Zap-70, the expression of Lck in CLL cells does not relate to disease prognosis [54,81,82]. In contrast, studies showed that Lck expression correlates with the sensitivity of CLL cells to pharmacological treatment and hence Lck is considered as a potential indicator of the response to CLL therapy and a target for the treatment of CLL [81,83]. Little is known on how Lck regulates BCR signaling. The BCR is composed of a plasma membrane anchored immunoglobulin recognizing antigens, which is associated with the signal transducing CD79a and CD79b chains. Signal transmission upon BCR crosslinking follows a molecular scheme similar to TCR triggering where the Src family kinase Lyn phosphorylates the ITAMs within CD79a and CD79b. This results in the recruitment and activation of the Syk family kinase Syk to the BCR, an event which is necessary for the assembly of a signalosome including the adaptor molecules BLNK and Grb2, the Tec family kinase Bkt and the Rho family GEF Vav, which is essential for the activation of PLCγ2 and the activation of more downstream signaling such as Akt, NF-kB and mitogen-activated protein kinases (MAPKs) [84]. Few studies have investigated the role of Lck in BCR signaling in detail. The data suggest that stronger BCR signaling intensity in CLL cells correlates with Lck expression [82,85]. These studies have also shown that Lck-mediated enhancement of BCR signaling correlates with a better survival and proliferation of CLL cells. In CLL cells, Lck associates with CD79a upon BCR engagement and functions in parallel to Lyn to phosphorylate CD79a and Syk (Figure 2) [73,82]. Indeed, inhibition of Lck activity using specific Lck inhibitors and, more importantly, siRNA-mediated suppression of Lck expression strongly reduced, but did not abolish BCR-mediated CD79a phosphorylation [82]. Lck also regulates downstream signaling leading to the activation of Erk1/2, Akt, and NFκB [82]. How Lck regulates more downstream BCR signaling pathways is not yet clear. It is possible that Lck plays a role in the assembly of the BLNK/Btk/Grb2 signalosome, which is required for the activation of PLCγ2 (indeed Lck regulates PLCγ2 in acute lymphoblastic leukemia, see below) or that Lck regulates PLCγ2 activation via alternative aberrant pathways. In this regard, it was shown that some CLL cells express SLP-76, a T-cell specific molecule required for the activation of PLCγ1, and that Lck is involved in the phosphorylation of SLP-76 in CLL cells [86]. Suppression of SLP-76 results in the inhibition of PLCγ2 phosphorylation and NFκB activation. Therefore, Lck may control the activation of PLCγ2 via SLP-76. Finally, a recent study has shown that B cells from CLL patients expressing high levels of Lck (but not in those where Lck was undetectable or expressed at low levels) display elevated basal phosphorylation of CD79a, Akt, and Erk1/2 [85]. Enhanced BCR signaling correlated with the expression of the activation markers CD69, CD38 and the proliferation marker Ki-67.

### 5.2. Acute Lymphoblastic Leukemia (ALL) of the B-Cell Compartment

Acute lymphoblastic leukemia is one of the most common forms of leukemia in children, which also affects adults [87]. ALL is characterized by malignant transformation and proliferation of lymphoid progenitor cells of either the B- or T-cell compartments. The majority (75%–85%) of ALL develops from precursors of the B-cell lineage (B-ALL). Chromosomal aberrations are the hallmark of ALL, which, however, are not sufficient to generate leukemia. Additional factors contribute to the development of the disease, including kinase-activating mutations as well as mutations in key transcription factors involved in B-cell development. Lck was found to be expressed in B-ALL and its ectopic expression appears to depend on aberrant activity of translocated transcription factors. One study has shown that Lck overexpression in the B-cell precursor form of ALL (BCP-ALL) seems to correlate with translocations of the *PAX5* gene both in human and mouse leukemic cells [88]. In addition to be overexpressed, Lck was found to be hyperactivated. Lck hyperactivation in patients with BCP-ALL correlates with poor clinical response to prednisone [89]. The reason why Lck is hyperactivated appears to be due to a reduction in the expression of Csk and a concomitant reduced phosphorylation of the inhibitory Y505 [88]. In BCP-ALL, Lck also seems to function downstream of the IL-7R to activate STAT5 and its downstream target genes *cMYC* and *CCND2*, as an Lck inhibitor attenuated STAT5 signaling in leukemic cells (Figure 2) [88]. Similarly, in a form of BCP-ALL harboring a chromosomal translocation coding for the chimeric transcription factor E2A-PBX1, it was found that Lck, together with Zap-70 and Syk, was overexpressed [90]. Also, in this case the activity of the chimeric E2A-PBX1 transcription factor appeared to be involved in the expression of Lck. Additionally, it was shown that shRNA-mediated suppression of Lck expression decreases the phosphorylation of PLCγ2, which has a pathogenic role in this leukemic form, while inhibition of Lck in preclinical studies reduces leukemic cell growth.

### 5.3. Acute Myeloid Leukemia (AML)

AML is a genetically heterogeneous malignant disorder characterized by the clonal expansion and differentiation arrest of hematopoietic progenitor cells of the myeloid lineage [91]. From a clinical point of view, the outcome of the disease remains poor for the majority of patients and more recently new targeted agents have received US Food and Drug Administration (FDA) approval for the treatment of AML [92]. Little is known about the function of Lck in AML. Initial studies indicated high expression of Lck in leukemic cells from patients with less differentiated AML, i.e., AML-0 and AML-1 [60]. Analysis of two AML cell lines of the M5-subtype, CTV-1 and THP-1, showed that THP-1 cells do not respond to various Lck inhibitors, whereas CTV-1 cells, which overexpress a mutated active Lck, are potently inhibited by several Lck inhibitors, including dasatinib [93]. Therefore, for patients with a similar oncogenic mechanism as in CTV-1 cells inhibition of Lck may be a potential therapeutic strategy. Several other studies also revealed that Lck functions both as an important signaling molecule and as a therapeutic target in AML [93,94]. Marhall and coworkers reported that Lck plays a role in AML expressing a constitutively active mutant of the cytokine receptor FLT3, FLT3-ITD, which displays an internal tandem duplication leading to constitutive signaling of the kinase [95]. Using Ba/F3 cells lacking endogenous Lck as a murine model for AML, it was shown that ectopic expression of Lck significantly enhanced the colony forming capacity of the FLT3-ITD mutant, but it had no effect on the survival or apoptosis of Ba/F3 cells transfected with FLT3 WT. FLT3-ITD^+^ cells expressing Lck also showed enhanced proliferative capacity and developed tumors earlier than cells transfected with control vector. Lck expression enhanced FLT3-ITD-mediated STAT5 activation but did not affect FLT3-ITD-induced Akt, Erk1/2 or p38 phosphorylation (Figure 2). Despite the fact that Lck appears to interact with FLT3/ITD, it remains unclear how Lck enhances FLT3/ITD-mediated signaling [95]. The potentiating effect on FLT3-ITD-mediated signaling is not limited to Lck as overexpression of the Src-family kinase (SFK) Fyn in FLT3-ITD^+^ Ba/F3 cells induced similar effects as observed for Lck [96]. Interestingly, SFK inhibitors reduced the viability of FLT3-ITD^+^ murine AML cells, thus suggesting a therapeutic role for this class of inhibitors in the treatment of the disease [95]. Another study has shown the beneficial effects of SFK inhibitors for the treatment of AML patients carrying co-occurring FLT3-ITD and NUP98-NSD1 mutations, which is among the most unfavorable AML forms associated with poor prognosis [97]. FLT3-ITD^+^/NUP98-NSD1^+^ patient cells express significantly higher levels of Bcl2A1 and the Src kinases Lck and Fgr compared to healthy CD34^+^ hematopoietic cells. Treatment of those patients with the SFK inhibitor dasatinib in combination with the Bcl2 inhibitor navitoclax was highly synergistic, thus representing a new clinically relevant treatment strategy [97].

### 5.4. Chronic Myeloid Leukemia (CML)

CML is a BCR-ABL(+) myeloproliferative disease which makes up 15–20% of all leukemia cases in adults. For those patients resistant or intolerant to BCR-ABL inhibitor imatinib, second- and third-generation tyrosine kinase inhibitors are successfully used in distinct CML disease states [98,99,100,101]. The SFK inhibitor dasitinib is approved for the treatment of all phases of CML and is first-line treatment for CML in the chronic phase [102]. Among SFKs, LYN and FYN appear to the major players in CML progression [103,104,105,106,107,108,109,110] although some reports found no role for SFK in CML [111,112]. With regard to Lck, nothing is known so far on its role in CML. Future analyses might nevertheless uncover unexpected functions of Lck in CML.

## 6. The Role of Lck in Tumors of Non-Hematopoietic Origin

Lck expression was detected in a number of solid cancers including breast cancer [113,114,115,116], colon cancer [117,118,119], and lung carcinoma [119,120,121]. These observations have led to the hypothesis that Lck may have cancer promoting functions and hence may represent a potential diagnostic biomarker and therapeutic target for solid cancers. Indeed, Lck inhibitors are used not only for the treatment of leukemia but also in various solid cancers [100,122].

The expression of Lck in patients suffering from cholangiocarcinoma is associated with tumor recurrence [123]. Both in vitro and in vivo models, treatment of cholangiocarcinoma cells with SFK inhibitor dasatinib showed tumor suppressive effects. In this form of cancer, the molecular pathomechanisms of Lck in tumor development have been investigated more closely. The transcriptional coactivator Yes-associated protein (YAP), which can be induced by PDGF receptor signaling and which plays a major role in the pathogenesis of cholangiocarcinoma [124], was found to be regulated by Lck (Figure 2) [123]. Lck phosphorylates YAP (on Y357) and both dasatinib and siRNA-targeted knockdown of Lck blocked YAP tyrosine phosphorylation. Moreover, treatment of cholangiocarcinoma cells with dasatinib induced YAP redistribution from the nucleus to the cytosol and downregulated expression of YAP target genes, which are involved in carcinogenesis [123].

Lck also seems to play a role in cancer stem cells (CSC) in endometrioid cancer models [125]. In this form of cancer, the cell surface complement inhibitor CD55 regulates self-renewal and cisplatin resistance in a complement-independent manner. Interestingly, CD55 signals via the transmembrane adaptor protein LIME (Lck-interacting transmembrane adaptor) (Figure 2) [125], which we have identified to interact with Lck [126]. Suppression of LIME expression in CSCs results in decreased levels of active Lck and also in impaired CD55-mediated signaling. Moreover, Lck plays a role in the upregulation of genes involved in DNA repair, including *MLH1* and *BRCA1*, and the resistance of CSCs to cisplatin. Indeed, treatment of these cells with the Fyn/Lck inhibitor saracatinib or suppression of Lck via shRNA sensitized CSCs to cisplatin [125].

Lck is also involved in cisplatin resistance of glioma cancer stem cells [127]. It has been proposed that radiation therapies used to target gliomas may expand the cancer stem cell population and, thereby increase the aggressiveness of tumors. Fractionated radiation caused a selective increase in the activity of Lck in glioma cells, whereas Lyn, Fyn, and other Src kinases were not significantly increased. The expansion of the glioma stem cell population (CD133^+^) induced upon fractionated radiation was significantly suppressed by siRNA-mediated knockdown of Lck but not by siRNAs against other Src family kinases (Lyn, Fyn, and Src). siRNA-mediated knockdown of Lck effectively restored the sensitivity of glioma cells to cisplatin and etoposide treatment. In addition, suppression of Lck inhibited the self-renewal capacity of glioma stem cells [127]. However, another study has shown that Lck is not required for the maintenance of glioma stem cells [128]. An additional function of Lck in glioma cells has been described in a recent study by Zepecki and co-workers [129]. They found strong expression of active Lck in human glioblastoma tissue sections. Application of an Lck inhibitor (A770041) at concentrations which inhibited Lck activity (but had no effect on the phosphorylation state of Src, Yes, Lyn, and Fyn) correlated with a decreased tyrosine phosphorylation of paxillin (Y118) and CrkII (Y221) selectively in pseudopodia [129] (Figure 2). Both paxillin and CrkII are involved in adhesion and migration and both proteins are deregulated in various cancer types [130,131]. Accordingly, Lck inhibition blocked the formation of pseudopodia and the migration of glioma cells in a human glioma cell-axon-oligodendrocyte co-culture model. In vivo administration of the Lck inhibitor in a glioma model reduced tumor size, whereas inhibition of Lck in glioma stem cells reduced self-renewal and tumor-sphere formation [129]. Collectively, these data suggest that by regulating migration, tumor growth and cancer stemness, Lck represents a novel important target in glioblastoma treatment.

Finally, Lck and also Lyn are overexpressed and active in several small cell lung cancer (SCLC) and non-small cell lung cancer (NSCLC) cell lines and lung cancer specimens from patients [132]. Dasatinib treatment blocked DNA synthesis and reduced cell numbers but it did not induce apoptosis in multiple lung cancer cell lines. The reason of the resistance to apoptosis seems to result from the fact that SFK inhibition induced autophagy. Indeed, when dasatinib treatment was combined with autophagy inhibitors robust apoptosis of lung cancer cells was induced.

## 7. Lck in the Nervous System

The major SFKs expressed in the brain are Src and FynB. They play a pivotal role in regulating neuronal differentiation and growth, myelinization, neurotransmitter signaling, synaptic plasticity, and behavior [133,134]. Although Lck was found to be expressed in neurons and throughout distinct regions of the brain, including the hippocampus, cerebellum and retina [135,136,137], the function of Lck in neurons and other cell types in the brain is rather unclear.

One brain cell type in which the function of Lck has been investigated is the microglia (the brain’s phagocytic compartment). Engagement of CD40 on microglia cells by CD40L induced Lck activity, Erk1/2 activation and TNFα production [138]. Microglia cells also express CD45 one of the regulators of Lck activity. Cross-linking of CD45 on resting microglia cells increased Lck activity consistent with the positive regulatory role of CD45 in the regulation of SFK. However, in microglia cells which were activated via CD40, CD45 ligation inhibited Lck activity, Erk1/2 activation and TNFα production. Thus, in activated microglia cells CD45 functions as an inhibitor of SFK [138].

Schwann cell proliferation, maturation and differentiation are dependent on the interaction of surface β1-integrins with the laminin matrix. Treatment of Schwann cells with laminin led to activation of Lck via β1-integrin [39]. Lck signaling involved the activation of paxilin, CrkII, and Rac-GTP and induced lamellipodia formation (Figure 2). Loss of Lck or the inhibition of Lck in Schwann cell root ganglion co-cultures showed a reduction in Schwann cell migration, myelin formation and internode length. Thus, Lck seems to be a regulator of cytoskeletal dynamics, migration and myelination in the peripheral nervous system [39].

One interesting observation regarding Lck in the brain is its expression in neurons of the hippocampus [135], which is critical for learning and memory. Indeed, more recently Lck was shown to be involved in the regulation of neuritic outgrowth and long-term hippocampal synaptic plasticity (long-term memory), but not in spontaneous excitatory synaptic transmission or short-term synaptic potentiation [139]. Accordingly, selective inhibition of Lck reduced hippocampus-dependent spatial learning and memory formation *in vivo.* In this line, using Lck siRNA and Lck-deficient mice, it was found that Lck plays an important role in neuronal preconditioning in cellular and animal models of stroke [140]. Cortical neurons preconditioned with oxygen deprivation or exposure to the N-methyl-D-aspartate (NMDA) receptor agonist NMDA showed enhanced Lck kinase activity. An Lck antagonist attenuated the neuroprotective effect of preconditioning in the mouse focal ischemia model. It was additionally found that PKCε is an upstream regulator of Lck and that Fyn is a target of Lck (Figure 2) [140]. Thus, the PKCε-Lck-Fyn signaling axis may be a target for the development of stroke therapies.

In earlier studies, Lck has been associated with Alzheimer disease, the most prevalent cause of dementia. Decreased levels of Lck were observed in the brains of Alzheimer patients and single nucleotide polymorphism of the Lck gene was detected in some cases of Alzheimer disease [141]. However, more recently it was found that Fyn is activated by aggregated brain amyloid-beta (Aβ) via interaction with cellular prion protein [142,143] and that Fyn phosphorylates the microtubule associated protein Tau (Y18) [144]. Since the deposition of Aβ-plaques and Tau filaments are considered as major factors in induction and treatment of Alzheimer disease, the research is focusing on Fyn, rather than Lck. Although Lck interaction with Tau has also been reported [145], Fyn has emerged as a prime target for preclinical therapeutic intervention with SFK inhibitor saracatinib in Alzheimer disease. To what extent saracatinib inhibits Lck functions in the brain under this therapeutic regimen is still unresolved [146].

## 8. Conclusions

Since the first report showing that Lck is specifically expressed in T lymphocytes, a lot of knowledge has been gained about the function of Lck in TCR signaling. In addition, it has become evident that Lck is expressed in other cell types and is involved in signaling downstream of other receptors. Nevertheless, the understanding of the precise molecular mechanisms of how Lck regulates these additional signaling pathways requires further investigations. It will be important to identify the substrates of Lck, how the activity of Lck is regulated in these cell types, and how inhibition of Lck will alter cell responses. In this regard, it will be essential to improve the specificity and efficacy of the currently available Lck inhibitors to increase the therapeutic options for the treatment of human diseases in which Lck plays a pathological role. Since Lck plays a role in cancer cell signaling as well as in T-cell function, it will be necessary to define therapeutic strategies to selectively target Lck in tumor cells without impairing the responses of tumor infiltrating lymphocytes. This is a critical issue common to other kinase inhibitors targeting signaling molecules expressed in both cancer and immune cells (e.g., B-Raf, AKT, mTOR inhibitors). Future research will most likely uncover additional exciting functions of Lck in other pathophysiological processes.

## Abbreviations

ALL	acute lymphoid leukemia
AML	acute myeloid leukemia
CAR	chimeric antigen receptor
CML	chronic myeloid leukemia
SFK	src family kinase
WT	wild-type
